# Intratumoral IL-28B Gene Delivery Elicits Antitumor Effects by Remodeling of the Tumor Microenvironment in H22-Bearing Mice

**DOI:** 10.1155/2022/1345971

**Published:** 2022-07-28

**Authors:** Zhi Li, Jianghua Wang, Chong Chen, Qi He, Xiaoying Xu, Zejiao Da, Bo Wang, Meng Wang, Xiaotong Gao, Guochao Zhang, Qi Gao, Xiaoli Si, Yanping Luo, Xingming Ma

**Affiliations:** ^1^Department of Immunology, School of Basic Medical Sciences, Lanzhou University, Lanzhou 730000, China; ^2^The Second Hospital of Lanzhou University, Lanzhou 730030, China; ^3^Key Lab of Preclinical Study for New Drugs of Gansu Province, Lanzhou 730000, China

## Abstract

IL-28B, belonging to type III interferons (IFN-*λ*s), exhibits a potent antitumor activity with reduced regulated T cells (Tregs) population, yet the effect of IL-28B on the tumor microenvironment (TME) and if IL-28B can downregulate Tregs directly *in vitro* are still unknown. In this study, we investigated the effects of IL-28B on Tregs in the spleen and TME in H22 tumor-bearing mice and verified the downregulation of IL-28B on Tregs *in vitro*. We found that rAd-mIL-28B significantly inhibited tumor growth and reduced the frequency of splenic CD4^+^Foxp3^+^ T cells. The levels of CXCL13, ICAM-1, MCP-5, and IL-7 in the serum, and the levels of IL-15 and sFasL in the tumor tissue decreased significantly after rAd-mIL-28B treatment relative to rAd-EGFP. Furthermore, the percentage of CD8^+^ cells in the TME was significantly increased in the rAd-mIL-28B group compared with the untreated group. *In vitro*, splenocytes were stimulated with anti-CD3/CD28 and IL-2 in the presence of TGF-*β* with or without IL-28B for three days and followed by flow cytometric, RT-PCR, and IL-10 production analysis. The results showed that IL-28B significantly reduced the proportion of induced Foxp3^+^ cells. It demonstrated that IL-28B may be used as a promising immunotherapy strategy against cancer.

## 1. Introduction

The tumor microenvironment (TME) is a comprehensive network in the local tumor environment, which is built up by multiple cell types including tumor cells, endothelial cells, stromal fibroblasts and tumor-infiltrating T lymphocytes, and cytokines/chemokines produced by different cells. TME has been identified as a crucial role in determining tumor progression and prognosis in different malignancies, and immunosuppression of TME is associated with immune escape [1].

The immunosuppressive TME is partially associated with regulated T cells (Tregs). Tregs, characterized by lineage-specific Foxp3 expression, are critical to suppress excessive immune responses to maintain homeostasis [2], however, in favor of tumor progression. Abundant accumulation of Tregs in tumor tissues may be due to the recruitment of Tregs from other parts of the body to the tumor area by chemokines and/or proliferation/differentiation of CD4^+^CD25^+^ Tregs from peripheral CD4^+^CD25^−^ T cells [3]. Tregs infiltrating tumors are correlated with poor prognosis in cancer patients [4]. Tregs play vital roles in tumor progression by suppressing effective T cell activity such as CD8^+^ T cells, which are key immune cells killing cancer cells [5, 6]. Reducing Tregs or altering their suppressive activity becomes a key immunotherapy strategy against cancer [7]. Moreover, the cytokines/chemokines produced by immune cells were thought to have dual roles in cancer development, depending on the TME [8]. Thus, TME modulation to kill tumor cells effectively in malignancies is essential for effective immunotherapy [9].

IL-28B, a member of the type III interferon (IFN-*λ*s) family, has potent antiviral infection, immunoregulatory effect, and antitumor activity. In the past few years, the effects of IL-28B on Tregs in vaccine immunity have been investigated. Morrow et al. [10] observed that IL-28B, as an adjuvant in DNA vaccination, reduced splenic Tregs and enhanced adaptive immunity in vaccinated mice. In our previous investigations, we also demonstrated that IL-28B downregulated Tregs population during tuberculosis subunit vaccine immunization [11]. The antitumor activity of IL-28B was associated with reduced splenic Tregs [12]; however, the effect of IL-28B on the TME and whether IL-28B downregulating Tregs directly *in vitro* remain unclear.

## 2. Materials and Methods

### 2.1. Preparation of Recombinant Adenovirus Vectors

The recombinant adenovirus vectors express IL-28B protein (rAd-mIL-28B), in which the mouse IL-28B gene was constructed by the pAd/PL-DEST adenovirus expression vector with the aid of the shuttle plasmid pYr-adshuttle-1, were obtained from our laboratory and confirmed by PCR analysis as reported previously [11]. After being infected with rAd-mIL-28B, the HEK293 cells suffered from freeze-thaw damage to release the adenovirus. Finally, adenoviral titers were measured by the method of Reed and Muench [13]. The constructed adenovirus vector encoding enhanced green fluorescent protein (rAd-EGFP), as the control of rAd-mIL-28B, was prepared as described above.

### 2.2. Animal and In Vivo Evaluation of Tumor Growth

Female BALB/c mice, 6-8 weeks old, were obtained from the experimental animal center of Gansu University of Chinese Medicine and maintained in specific pathogen-free (SPF) conditions with light-cycled and temperature-controlled. The mice could get food and water *ad libitum.* All experiments were approved by the Lanzhou University ethics committee and handled in compliance with the protocols and guidelines of the Institutional Animal Caring and Using Committee.

1 × 10^6^ H22 cells in 100 *μ*l PBS were inoculated subcutaneously into the right flank of the mice (day 0). When the tumor mass became palpable (approximately 5 mm in diameter), the mice were randomized and received intratumoral injections of 100 *μ*l 1 × 10^8^ plaque-forming units (PFUs) of rAd-mIL-28B or rAd-EGFP on days 4, 7, and 10, or intraperitoneal injections of cisplatin at a dose of 2.5 mg/kg on days 7, or without treatment. Cisplatin, a broad-spectrum and most active cytotoxic agent [14], was used as a positive control in the study.

The tumor was measured with a caliper in two perpendicular directions. Tumor volume was calculated using the formula: tumor volume = (smallest diameter)^2^ × (largest diameter) × 0.52. Mice with tumor size >1.0 × 1.0 cm were euthanized by injection of pentobarbital sodium (50 mg/kg) intraperitoneally.

### 2.3. Isolation of Lymphocytes from the Tumor Tissue and Spleen

The tumors and spleens from the H22-bearing BALB/c mice were harvested. The tumor-infiltrating lymphocytes were prepared as described with minor modification [15]. In brief, tumors were mechanically disaggregated, then cut into small pieces, and followed by digestion with 0.9 mg/ml collagenase I, 0.9 mg/ml collagenase IV, 0.9 mg/ml hyaluronidase and 1.5 mg/ml DNase I (all from Solarbio, Beijing, China) in Hanks' buffer for 1 h at 37°C. Then, the digestion was terminated by Hanks' buffer containing 5% FBS (Gibco, New York, USA). After the lysates were filtered through a 200-mesh nylon strainer with a plunger, cells were collected by centrifugation at 250 × g for 10 min at 4°C and then washed twice with PBS. The tumor-infiltrating lymphocytes were then prepared using density gradient centrifugation with mouse 1x lymphocyte separation medium (Dakewe Biotech, Shenzhen, China) according to the manufacturer's indications. Finally, cells were washed twice with RPMI 1640 and resuspended in the flow cytometry staining buffer (PBS buffer containing 0.1% NaN3 and 0.1% BSA) for flow cytometry analysis.

Lymphocytes from the spleen were prepared as described previously [16]. Briefly, the spleens of mice were removed aseptically, then ground with a sterile syringe plunger, and collected single-cell suspensions after passing through a sterile 200-mesh nylon strainer. Finally, the lymphocytes were isolated with mouse 1x lymphocyte separation medium from the cell suspension.

### 2.4. Flow Cytometric (FCM) Analysis

Cells were resuspended in flow cytometry staining buffer and incubated with FITC-conjugated anti-CD4, PerCP-conjugated anti-CD8, and PE-conjugated anti-PD-1 or PE-conjugated anti-CD25 (all from BD, New York, USA) at 4°C for 30 min in the dark for surface staining. For detecting Foxp3, cells were washed and then placed into the fixation/permeabilization buffer. Followed by centrifugation, cells were washed with flow cytometry staining buffer and stained with APC-conjugated anti-Foxp3 antibodies (both from eBioscience, San Diego, USA) as recommended by the manufacturer. Then, cells were washed and resuspended in flow cytometry staining buffer. Finally, the samples were performed using a NovoCyte flow cytometer (ACEA Biosciences, Hangzhou, China) and analyzed using the NovoCyte software.

### 2.5. Serum and Intratumoral Cytokine Analysis

The serum was collected and stored at -20°C for further analysis. Tumor tissues were cut and then lysed using the cell lysis buffer containing proteinase inhibitor cocktail (RayBiotech, GA, USA). Then, the lysate was placed in a shredder and followed by centrifugation at 14,000 rpm for 10 min. Finally, the supernatant was transferred to new tubes and kept at -20°C. The serum and the supernatant of the tumor homogenate were assessed using commercial mouse cytokine array 5 kits (RayBiotech, GA, USA). The assay measures 40 cytokines: bFGF, CXCL13, CD30L, Eotaxin-1, Eotaxin-2, Fas Ligand, GCSF, GM-CSF, ICAM-1, IFN*γ*, IL-1*α*, IL-1*β*, IL-2, IL-3, IL-4, IL-5, IL-6, IL-7, IL-10, IL-12p40, IL-13, IL-15, IL-17A, IL-21, CXCL1, Leptin, LIX, MCP-1, MCP-5, M-CSF, CXCL9, MIP-1*α*, MIP-1*γ*, CXCL4, RANTES, CCL17, CCL1, TNF*α*, TNFRI, and TNFRII. All procedures were conducted by the manufacturer's instructions. The signals were scanned and detected with the aid of an InnoScan 310 microarray scanner (Innopsys, Carbonne, France).

### 2.6. Cell Culture

Spleens were removed aseptically from the euthanized normal female BALB/c mice. The splenocytes were prepared with the lymphocyte separation medium as described above. Collected cells were washed twice with RPMI 1640 and resuspended in RPMI 1640 medium supplemented with 10% FBS, 100 IU/ml penicillin, and 100 *μ*g/ml streptomycin (Sigma-Aldrich, MO, USA) for culturing experiments. Cell viability was evaluated by the trypan blue dye exclusion test.

Splenocytes (5 × 10^6^ cells/ml) in quintuplicate were seeded in 96-well plates supplemented with RPMI 1640 complete medium in a final volume of 200 *μ*l. The cells were cultured with anti-CD3/anti-CD28 antibody (Abs) (both from eBioscience, San Diego, USA), IL-2 (PeproTech, London, USA), and transforming growth factor-*β* (TGF-*β*) (R&D Systems, MN, USA) in the presence or absence of IL-28B (5, 50, and 400 ng/ml) (R&D Systems, MN, USA) were added to the culture medium. Three days later, cells were harvested and analyzed for CD4, CD25, and Foxp3 expression by flow cytometry.

### 2.7. ELISA and qRT-PCR Analysis

Splenocytes (5 × 10^6^ cells/ml) in triplicate were cultured in 24-well plates supplemented with the RPMI 1640 complete medium in a final volume of 1 ml at 37°C, 5% CO_2_. Splenocytes were stimulated with anti-CD3 (500 ng/ml), anti-CD28 (500 ng/ml), and IL-2 (5 ng/ml) in the presence of TGF-*β* (1.25 ng/ml) and/or 50 ng/ml IL-28B. After 3 days, the cell-free culture supernatants were collected and assessed for IL-10 production using the commercial ELISA kit (MultiSciences, Hangzhou, China) according to the manufacturer's instructions. Total RNAs were extracted from cultured cells using a TRIzol reagent (TaKaRa Bio Inc., Dalian, China) as directed by the manufacturer. cDNAs were synthesized by reverse transcription with a PrimeScript™ RT reagent Kit with gDNA Eraser (TaKaRa Bio Inc., Dalian, China) and then subjected to PCR amplification with SYBR® Premix Ex Taq™ II kit (TaKaRa Bio Inc., Dalian, China). Real-time PCR for Foxp3 while the endogenous control glyceraldehyde-3-phosphate dehydrogenase (GAPDH) was performed with a CFX96 Real-Time PCR system (Bio-Rad, CA, USA).

The thermal cycling parameters were as follows: stage 1, 95°C for 10 s; stage 2, 40 cycles of 95°C for 10 s, 60°C for 15 s, and 72°C for 30 s; and stage 3, 95°C for 10 s, 65°C for 1 min, 97°C for 10 s.

Primer sequences for Foxp3 and GAPDH were as follows: Foxp3-forward: 5′-TGCCTTCAGACGAGACTTGGA-3, Foxp3-reverse: 5′-GGCATTGGGTTCTTGTCAGAG-3; GAPDH-forward: 5′-AAATGGTGAAGGTCGGTGTGAAC-3, GAPDH-reverse: 5′-CAACAATCTCCACTTTGCCACTG-3.

The relative mRNA expression of Foxp3 was normalized to the housekeeping gene GAPDH and analyzed using the 2^−∆∆CT^ method [17]. All samples were performed in triplicate.

### 2.8. Statistical Analysis

The one-way analysis of variance (ANOVA) was used to determine the statistical significance (*P* < 0.05) between multiple groups. The data were analyzed using SPSS software version 20.0 and expressed by mean ± SD. The plotting functions of GraphPad Prism version 6.0 were used.

## 3. Results

### 3.1. Intratumoral IL-28B Gene Transfer Suppressed H22 Tumor Growth

**Figure 1:**
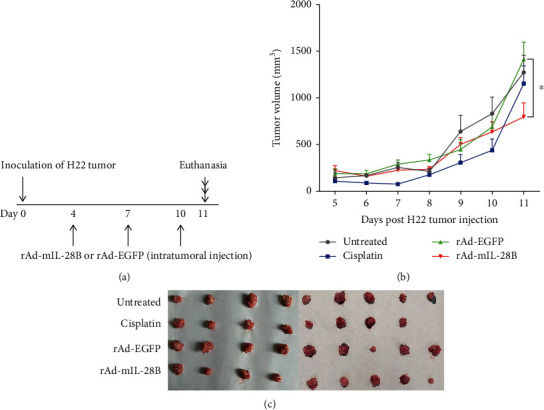
Intratumoral IL-28B gene transfer reduced tumor growth in a subcutaneous H22 hepatoma model. (a) Schema of the experiment was shown. (b) Tumor growth measurements. Data were shown as mean ± standard deviation (SD). ^∗^*P* < 0.05 for rAd-mIL-28B vs. rAd-EGFP. (c) Representative image of detached tumors. Data from one experiment, each performed with nine mice, are shown.

To investigate the potential antitumor effect of IL-28B, H22-bearing mice were treated with rAd-mIL-28B as described in methods and Figure 1(a). There was no significant difference in body weight among all groups (data not shown). The mean tumor volume in the rAd-mIL-28B group (796.74 ± 447.58 mm^3^) reduced compared with that in the rAd-EGFP group (1415.27 ± 513.37 mm^3^) (*P* < 0.05) (Figure 1(b) and Table 1). Besides, the tumor volumes of untreated and cisplatin groups were 1275.19 ± 544.43 mm^3^ and 1150.75 ± 552.23 mm^3^, respectively (Table 1). The size of tumors in the cisplatin group did not increase early. But the tumors grow rapidly after having been inoculated for 7 days in Figure 1(b). The picture of tumor tissue retrieved from each group was shown in Figure 1(c). And the mean tumor weight in the rAd-mIL-28B-treated group (0.69 ± 0.34 g) decreased compared with the rAd-EGFP-treated group (1.05 ± 0.45 g, tumor inhibition rate 34.07%) (*P* < 0.05) (Table 1). Furthermore, the mean tumor weight in the cisplatin group and untreated group was 0.87 ± 0.41 g and 0.95 ± 0.49 g, respectively (tumor inhibition rate 17.90%) (Table 1).

### 3.2. Intratumoral IL-28B Gene Transfer Reduced the Frequency of Splenic CD4^+^PD-1^+^ T Cells and CD4^+^Foxp3^+^ Tregs

**Figure 2:**
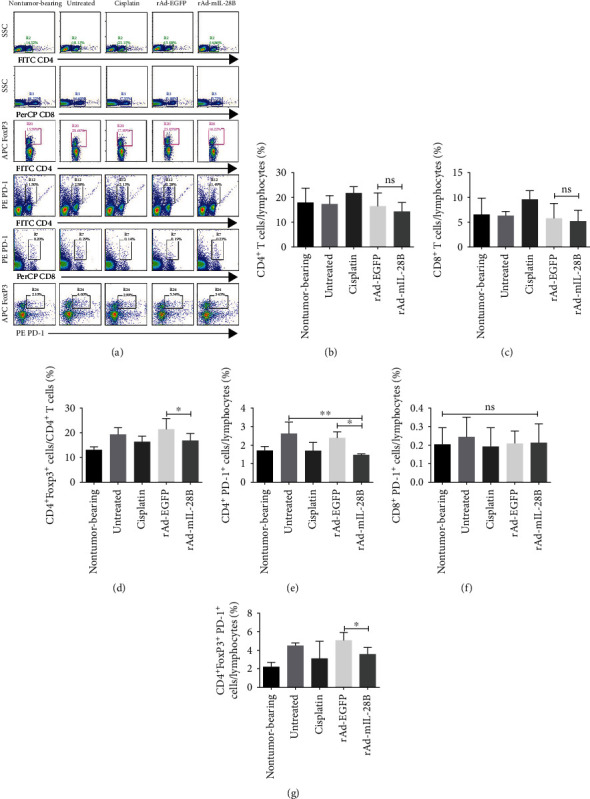
Intratumoral IL-28B gene transfer decreased the frequency of Tregs in the spleen. Spleens were harvested; processed into single-cell suspension; stained with FITC-conjugated anti-CD4, PerCP-conjugated anti-CD8, PE-conjugated anti-PD-1, and APC-conjugated anti-Foxp3; and analyzed by flow cytometry. (a) Representative dot plots and percentages of cells bounded by each region are shown in panels. (b) Statistical analysis of the percentages of CD4^+^ T cells, CD8^+^ T cells, and CD4^+^Foxp3^+^ cells among CD4^+^ T cells, CD4^+^PD-1^+^ T cells, CD8^+^PD-1^+^ T cells, and CD4^+^ Foxp3^+^ PD-1^+^ T cells in the spleens were shown. Data from one experiment, each performed with three mice, are shown. ^∗^*P* < 0.05, ^∗∗^*P* < 0.01; and ns indicated *P* > 0.05.

The flow cytometric scatter plot analysis were shown in Figure 2(a). The frequencies of CD4^+^ T cells and CD8^+^ T cells in the spleen (Figures 2(b) and 2(c)) did not show a statistical difference between the rAd-mIL-28B and rAd-EGFP groups. Compared with rAd-EGFP, the frequency of CD4^+^Foxp3^+^ among CD4^+^ T cells in the spleen was significantly reduced in the rAd-mIL-28B group (Figure 2(d)). The percentage of splenic CD4^+^PD-1^+^ T cells in the rAd-mIL-28B group decreased compared with the rAd-EGFP group or the untreated group (Figure 2(e)). There was no significant difference in splenic CD8^+^PD-1^+^ T cells among all groups (Figure 2(f)). Furthermore, the percentage of CD4^+^Foxp3^+^PD-1^+^ T cells decreased significantly in mice treated with rAd-mIL-28B compare with rAd-EGFP-treated mice (Figure 2(g)).

### 3.3. Intratumoral IL-28B Gene Transduction Increased the Tumor-Infiltrating CD8^+^ T Cells

**Figure 3:**
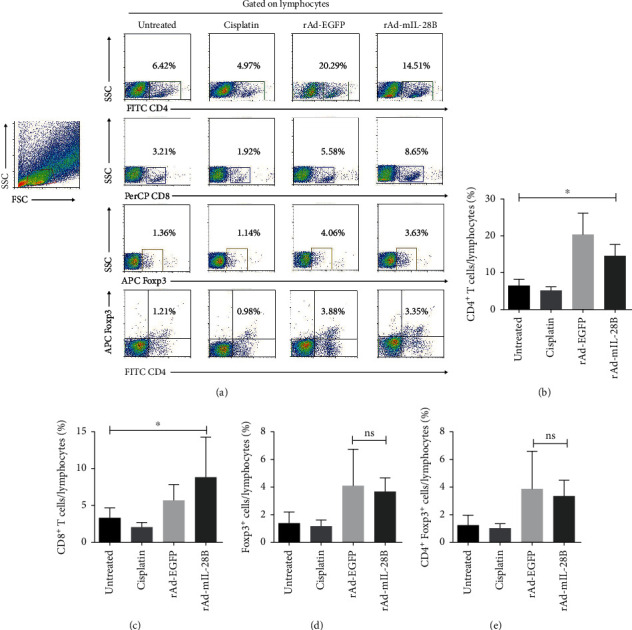
Intratumoral IL-28B gene transfer did not change the tumor-infiltrating regulatory T cells. The frequencies of CD4^+^, CD8^+^, Foxp3^+^, and CD4^+^Foxp3^+^ cells in TME. (a) Representative dot plots and percentages of cells bounded by each region are shown in panels. (b) Statistical analysis of the percentages of CD4^+^, CD8^+^, Foxp3^+^, and CD4^+^Foxp3^+^ cells in TME were shown (below panels). Data from one experiment, each performed with three mice, are shown. ^∗^*P* < 0.05; ns indicated *P* > 0.05.

To analyze whether rAd-mIL-28B affected the tumor immunotolerant microenvironment, the frequencies of CD4^+^, CD8^+^, Foxp3^+^, and CD4^+^Foxp3^+^ T cells in tumor tissure were examined by flow cytometic analysis. The flow cytometric scatter plot analysis was shown (Figure 3(a)). Remarkably, rAd-mIL-28B led to a massive increase in CD4^+^ T and CD8^+^ T cell populations compared with the untreated group in TME (Figures 3(b) and 3(c)). The proportion of CD8^+^ T cells showed an upward trend without reaching statistical significance in the rAd-mIL-28B group compared with the rAd-EGFP group. Furthermore, the mainly increased T cells in the TME induced by rAd-mIL-28B treatment were CD8^+^ T cells. However, the frequencies of Foxp3^+^ or CD4^+^Foxp3^+^ T cells in TME had no significant difference in the rAd-mIL-28B-treated tumors compared with that in rAd-EGFP-treated tumors (Figures 3(d) and 3(e)).

### 3.4. Il-28B Altered Peripheral and Local Cytokine/Chemokine Production *In Vivo*

Cytokine levels in the serum and tumor tissue, respectively, reflected a systemic and localized reaction to a tumor [18]. To assess changes in the inflammatory environment, we measured cytokine/chemokine levels in serum and tumor tissues.

**Figure 4:**
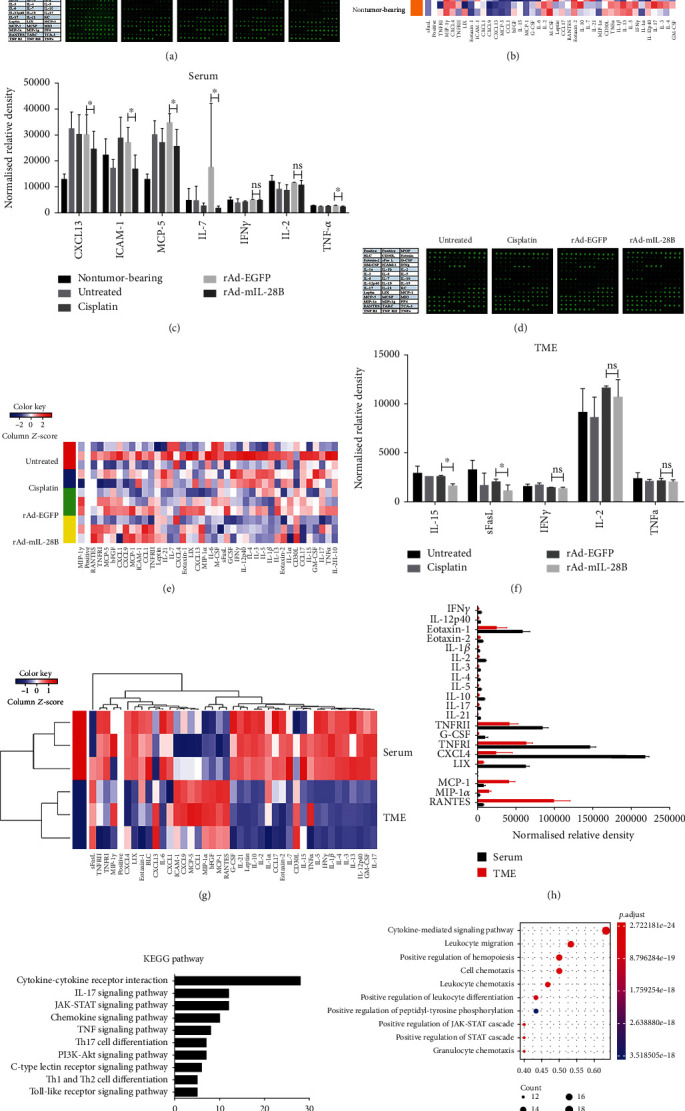
The peripheral and local cytokines production altered by IL-28B in vivo. Serum and tumor tissues were harvested and analyzed the cytokines by use of a commercial quantibody mouse cytokine array 5. The array images (a), a heat map of cytokine expression (b), and the mean signals strength of CXCL13, ICAM-1, MCP-5, IL-7 IFN-*γ*, IL-2, and TNF-*α* in the serum (c) were shown. The array images (d), a heat map of cytokine expression (e), and the mean signals strength of IL-15, sFasL, IFN-*γ*, IL-2, and TNF-*α* in TME (f) were shown. The mean signals strength derived from quadruplicate samples (*n* = 3). The hierarchical cluster analysis are used to analyze dissimilarities between serum and tumor tissues of rAd-mIL-28B (g). The moderated *t*-statistic was used for significance analysis to screen the differential proteins, which were defined as those with adjusted *P* < 0.05 and fold change > 1.2 or fold change < 1.2. Red indicates higher expressions and green indicates lower expressions. The levels of cytokines which changed more than twofold in serum and tumor tissues were shown (h). The KEGG pathway analysis (i) and Gene Ontology- (GO-) based biological process annotation of the differentially expressed proteins (j) were shown. Data from one experiment, each performed with three mice, are shown. TME: tumor microenvironment. ^∗^*P* < 0.05; ns indicated *P* > 0.05.

The array images (Figure 4(a)) and a heat map of cytokine expression (Figure 4(b)) in serum were shown. The expressions of serum CXCL13, ICAM-1, MCP-5, IL-7, and TNF-*α* in tumor-bearing mice treated with rAd-mIL-28B were lower compared to rAd-EGFP-treated mice (*P* < 0.05) (Figure 4(c)). There was no significant difference in the level of IFN-*γ* and IL-2 in the serum between the rAd-mIL-28B and rAd-EGFP groups (Figure 4(c)).

The array images (Figure 4(d)) and a heat map of cytokine expression (Figure 4(e)) in tumor tissues were shown. The levels of intratumoral IL-15 and sFasL decreased in the rAd-mIL-28B group relative to those in the rAd-EGFP group (Figure 4(f)). There was no significant difference in the level of IFN-*γ*, IL-2, and TNF-*α* in TME between the rAd-mIL-28B and rAd-EGFP-treated groups (Figure 4(f)).

The differentially expressed proteins (DEP), which were defined as those with adjusted *P* < 0.05 and fold change > 1.2 or fold change < 1.2, between the serum and tumor tissues in the rAd-mIL-28B group were obtained and shown in a hierarchical clustering (Figure 4(g)). Compared with tumor tissues, the level of IFN*γ*, IL-12p40, Eotaxin-1, Eotaxin-2, IL-1*β*, IL-2, IL-3, IL-4, IL-5, IL-10, IL-17, IL-21, TNFRII, G-CSF, TNFRI, CXCL4, and LIX increased in the serum over twofold after rAd-mIL-28B treatment (Figure 4(h)). Meanwhile, rAd-mIL-28B induced an increased expression of RANTES, MIP-1*α*, and MCP-1 higher twofold in tumor tissues than that in serum (Figure 4(h)). It was identified that the cytokine-cytokine receptor interaction (28 DEP), IL-17 signaling pathway (12 DEP), JAK-STAT signaling pathway (12 DEP), chemokine signaling pathway (10 DEP), TNF signaling pathway (8 DEP), Th17 cell differentiation (7 DEP), PI3K-Akt signaling pathway (7 DEP), C-type lectin receptor signaling pathway (6 DEP), Th1 and Th2 cell differentiation (5 DEP), and Toll-like receptor signaling pathway (5 DEP) were significantly enriched between tumor tissues and serum in the KEGG pathway analysis (Figure 4(i)). The DEPs are mainly related to the cytokine-cytokine receptor interaction via KEGG pathway analysis. In consistent with the finding, the Gene Ontology- (GO-) based biological process analysis showed the cytokine-mediated signaling pathway was mainly associated with differential protein expression in serum and cancer tissues (Figure 4(j)).

### 3.5. Il-28B Reduced the Generation of Foxp3^+^ iTreg Cells *In Vitro*

**Figure 5:**
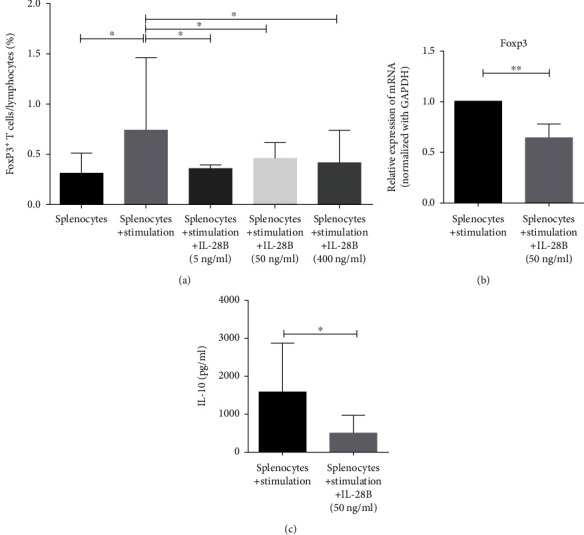
IL-28B inhibited the proportion of Foxp3^+^-producing cells in a dose-dependent manner concomitant with a reduction in IL-10 production and Foxp3 mRNA expression. Splenocytes isolated from normal BALB/C mice were stimulated with anti-CD3/CD28 mAbs supplemented with IL-2 in the presence of TGF-*β*±IL-28B (5, 50, and 400 ng/ml). Splenocytes cultured in complete RPMI 1640 medium were used as the control. Cells stimulated with 500 ng/ml anti-CD3/CD28 mAbs, 5 ng/ml IL-2, and 1.25 ng/ml TGF-*β* were defined as the stimulation group. After three days, cells were harvested for flow cytometry and RT-PCR analysis, and cell-free culture was collected for IL-10 assay. (a) The percentages of Foxp3^+^ T cells were shown in bar graphs. Data were from six independent experiments. (b, c) The Foxp3 mRNA expression and IL-10 level were shown. Data from one experiment, each performed with three samples, are shown. ^∗^*P* < 0.05; ^∗∗^*P* < 0.01.

Splenocytes were isolated from mice stimulated with anti-CD3/CD28 and IL-2 in the presence of TGF-*β* for three days and analyzed the expression of Foxp3 by flow cytometry. The results have shown that the proportion of Foxp3^+^ cells increased significantly in the splenocyte stimulation group compared with the splenocyte group (Figure 5(a)). After adding IL-28B at 5, 50, or 400 ng/ml, the percentage of Foxp3^+^ cells was significantly reduced than the splenocyte stimulation group in the absence of IL-28B (Figure 5(a)).

The expression of Foxp3 mRNA decreased significantly when 50 ng/ml IL-28B was added than the splenocyte stimulation group (Figure 5(b)). Compared to the splenocyte stimulation group, the concentration of IL-10 in the media was lower in the presence of IL-28B (50 ng/ml) than the splenocyte stimulation group (Figure 5(c)).

## 4. Discussion

To investigate the effect of IL-28B on the TME and test whether IL-28B downregulating Tregs directly *in vitro*, rAd-mIL-28B was injected into H22 tumor-bearing mice and the effect of IL-28B on iTregs *in vitro* was also studied. The results showed that rAd-mIL-28B decreased the volume and weight of the tumors, reduced the frequency of splenic CD4^+^Foxp3^+^ and CD4^+^PD-1^+^ T cells, and lower levels of CXCL13, ICAM-1, MCP-5 and IL-7 in serum compared to that in the rAd-EGFP group. Meanwhile, the expression of intratumoral IL-15 and sFasL were lower in the rAd-mIL-28B group than the rAd-EGFP group. The proportion of CD8^+^ cells in the TME was significantly elevated in the rAd-mIL-28B group compared with the untreated group. The expressions of RANTES, MIP-1*α*, and MCP-1 were more than two times higher in tumor tissues than in he serum after rAd-mIL-28B treatment. Furthermore, we found the percentage of Foxp3^+^ cells decreased significantly when splenocytes were stimulated with anti-CD3/CD28, IL-2, and TGF-*β* with IL-28B versus that without IL-28B *in vitro*.

IL-28B plays important antiviral and immunomodulatory activities [19]. Recently, the antitumor effects of IL-28B have been found. Numaski et al. [20] reported that the growth of IL-28-gene-transfected fibrosarcoma mice was suppressed by enhanced cytotoxic T lymphocyte (CTL) activity, augmented natural killer (NK) cells, and increased IFN*γ* production. The finding suggests that innate and adaptive immune responses play a critical role in IL-28B-medicated antitumor action. Consistent with this notion, the restrained rates of head and neck squamous cell carcinoma in mice treated with recombinant IL-28B and cisplatin were thought to be associated with tumor-specific CTL-mediated and NK-medicated antitumor activities [21]. Recently, Majumder et al. [22] reported that the number of lung tumor nodules was decreased along with enhanced CD8^+^ CTL activity in lung carcinogenesis-bearing mice injected with IL-28B alone or in combination with IL-27. We found that the tumor volume was decreased along with CD8^+^ cells trending to increased in TME in H22 hepatocellular carcinoma-bearing mice administered with rAd-mIL-28B. Considering what has been discussed above, we speculate that the antitumor effect of rAd-mIL-28B is presumably related to restore CD8^+^ T cell function.

Numerous studies reported that Tregs have been implicated in tumor immune escape via several mechanisms. For example, Tregs suppress the activation and expansion of tumor antigen-specific T cells. Tregs also inhibit antigen-presenting cells (APCs) to inhibit the priming and/or activation of conventional T cells. Tregs can also consume and compete with conventional T cells to limit the available IL-2 to conventional T cells. Interestingly, Tregs kill effector T cells directly through granzymes- and perforin-mediated cytotoxicity. Tregs can secrete immunosuppressive cytokines to downregulate APCs and effector T cell activity and regulate metabolism to prevent optimal T cell activation [2]. Tregs play vital roles in tumor progression by restraining different types of effector lymphocytes such as CD8^+^ CTLs and CD4^+^ T cells [6]. We found that IL-28B gene delivery significantly decreased H22 hepatocellular carcinoma growth and reduced the frequency of splenic CD4^+^Foxp3^+^ T cells, which is consistent with a previous report described by Morrow et al. [10]. Furthermore, the percentage of CD8^+^ cells in TME was significantly increased in the rAd-mIL-28B group compared with the untreated group. Previous research also reported that the repressive capacity of CD8^+^ T cells in lung carcinogenesis-bearing mice was recovered after IL-28B treatment [21]. Thus, our results indicated that rAd-mIL-28B may downregulate Tregs, recovering the frequency of CD8^+^ T cells to prevent tumor growth. TME is diverse and facilitates Tregs accumulation. Nishikawa et al. found that elaborated Tregs were abundant in tumor tissues accounting for 10-50% versus 2–5% of all CD4^+^ T cells in the peripheral blood of individuals without cancer [3]. The enhanced migration and proliferation activities led to Treg enrichment in the TME. One potential explanation is the selective trafficking of Tregs from the periphery by multiple chemokines produced by macrophages and/or tumor cells in TME. For example, tumor cells and tumor-associated macrophages secrete CCL22 facilitate Tregs accumulated. The second possibility is in situ conversion of Tconvs to Tregs in the TME and enhanced local proliferation. Tumors can contain a subset of immature dendritic cells that promote the activation and/or proliferation of Treg cells in a TGF-*β*-dependent manner in animal models. The large number of tumor or self-antigens presented by tumor cells contribute to the strong activation of Treg cells in the TME [2].

IL-28B reduced the frequency of splenic CD4^+^Foxp3^+^ cells in H22 tumor-bearing mice and the percentage of Foxp3^+^ cells after adding IL-28B at 5, 50, or 400 ng/ml. We also found that the frequency of CD4^+^Foxp3^+^ among CD4^+^ T cells in the spleen was reduced in the rAd-mIL-28B group compared with rAd-EGFP (16.78 ± 2.93 vs. 21.47 ± 4.17). The frequencies of CD4^+^Foxp3^+^ T cells in TME were 3.35 ± 1.15 vs. 3.88 ± 2.72 in the rAd-mIL-28B-treated tumors compared with that in rAd-EGFP-treated tumors. The downregulation effect of IL-28B on splenic Tregs, however, was not very strong. Tregs in the TME tend to aggregate more easily than those in the spleen. The large number of tumor or self-antigens presented by tumor cells contribute to strong activation of Treg cells in the TME [2]. Thus, the modest downregulation of rAd-mIL-28B on Tregs did not counteract the Tregs that accumulate continuously in the TME.

Programmed death-1 (PD-1), a negative costimulatory receptor, expresses on various cell types including activated T cells as well as CD4^+^ Treg cells. PD-1 interacts with its ligands which are highly expressed on tumor cells and T cells to dampen the proliferation and survival of T cells and induce tumor-specific T cells apoptosis [23]. Thus, PD-1 has become an important potential therapeutic target in tumors [24]. Furthermore, PD-1-PD-L1 ligation on CD4^+^ Tregs supported Treg stability and expansion [25]. The role of PD-1 on CD4^+^ Treg cells was strongly associated with high-risk prognostic factors in breast cancer patients, suggestive of synergistic contribution to impair the antitumor immune response [25]. We found that the percentages of splenic CD4^+^PD-1^+^ T cells and CD4^+^ Foxp3^+^PD-1^+^ T cells were lower in the rAd-mIL-28B group than the rAd-EGFP group, indicating rAd-mIL-28B may restore T cell function for its antitumor activity.

Antitumor-specific immunity involves a variety of cytokines that control the balance between tumor rejection by antigen-specific effector cells and suppressor mechanisms that allow tumors to escape the immune system [26]. The chemokine CXCL13, initially identified as chemoattractant for B cells, and its receptor drives hepatocellular carcinoma progression by activating the Wnt/B-catenin pathway [27]. Blockade of ICAM-1 reduced tumor incidence and metastasis *in vivo* [28]. Chemokine MCP-5 (CCL12) facilitates tumor metastasis and blockade of CCL2/CCR2 axis enhances CD8^+^ T cell-mediated antitumor immunity [29]. It has been reported that IL-7 shows antitumor activity in mice and humans through modulating CD8+ and CD4+ T cells [30]. However, Liu et al. reported that IL-7 may be related to protumor effects by preventing apoptosis in lung cancer cells [31]. Taken collectively, these results suggest that the cytokine-promoting tumor progression decreased in H22-bearing mice after rAd-mIL-28B treatment may be related to the antitumor effect of rAd-mIL-28B.

Cytokines are crucial for the recruitment and activation of specific leukocyte subsets within TME and are highly related to cancer prognosis [32, 33]. Kim et al. reported that cytokine levels in tumor tissue reflected a localized reaction to a tumor [18]. Multiple studies have implicated that the expression of IL-15 is crucial for antitumor responses via the augment of T cells and NK cells. However, it was recently reported that high IL-15 serum levels in melanoma patients were correlated with poor prognosis [34] and a lower level of IL-15 was more protective reported by Shirley SA et al. [35]. These contradictory reports may be related to different animal tumor models. Soluble FasL (sFasL), the cleavage product of FasL, induces an apoptotic cascade in target cells expressing Fas receptors. sFasL plays a critical role in immune evasion by inducing activated lymphocyte apoptosis [36], and the increased expression of sFasL is involved in tumor progression in solid tumors [37]. TGF-*β*, an effector molecule of Treg, can induce FasL expression in tumor cells [37]. We found that rAd-mIL-28B reduced the expression of intratumoral sFasL and increased CD8^+^ T cells in TME. As such, we interpret our findings that rAd-mIL-28B may decrease the levels of intratumoral sFasL, further to downregulate activated lymphocytes apoptosis such as CD8^+^ T cells.

Th1 cytokines, including IL-2, IFN-*γ*, and TNF-*α*, have important roles in antitumor immunity, whereas rAd-mIL-28B did not affect Th1 cytokines intumor, which may be due to rAd-mIL-28B mainly affected CD8^+^ T cells but not CD4^+^ cells.

We also found significant changes in systemic and TME cytokine response in tumor-bearing mice after treated with rAd-mIL-28B. Consistent with the results, Ray et al. [38] also found that the concentrations of cytokines between the serum and the TME were different. Except for trafficking immune cells to inflammation sites, chemokines also play vital roles in T cell-mediated antitumor immune responses such as RANTES, which attracts immunocytes to tumor tissues and exert antitumor efficacy [39]. Macrophage inflammatory protein-1*α* (MIP-1*α*), which recruits dendritic cells to the TME to enhance antitumor activity [40], MCP-1, shows tumor-suppressing activity by attracting and activating mononuclear cells [41]. After rAd-mIL-28B-treatment, higher levels of RANTES, MIP-1*α*, and MCP-1 in tumor tissues than in serum may be associated with tumor-restraining activity.

Morrow et al. speculated that the ability of IL-28B to reduce Tregs may through the IL-28 receptor [10]. To verify if there is a direct effect between IL-28B and Tregs, the effect of IL-28B on iTregs *in vitro* was studied. It is well accepted that IL-2, TGF-*β*, T cell receptor activation, and costimulation signals play important roles in the generation, conversion, survival, and suppressive activity of iTregs [42]. We induced iTregs verified by flow cytometric Foxp3 mRNA expression and IL-10 production analysis and found that IL-28B inhibited Foxp3^+^-producing cells stimulation group *in vitro*, suggesting that IL-28B may have a direct role on Tregs.

## 5. Conclusions

In conclusion, IL-28B elicits potent antitumor activity by downregulating Tregs, elevating the frequency of CD8^+^ T cells, and leading to a fall in the levels of protumor cytokines in TME. It demonstrated that IL-28B may be used as a promising immunotherapy strategy against cancer.

## Figures and Tables

**Table 1:** Intratumoral IL-28B gene transfer reduced tumor growth in a subcutaneous H22 hepatoma model.

Group	Tumor volume (mm^3^)	Tumor weight (g)	Tumor inhibition rate (%)
Untreated	1275.19 ± 544.43	0.95 ± 0.49	—
Cisplatin	1150.75 ± 552.23	0.87 ± 0.41	17.90
rAd-EGFP	1415.27 ± 513.37	1.05 ± 0.45	—
rAd-mIL-28B	796.74 ± 447.58^∗^	0.69 ± 0.34^∗^	34.07

The tumor volume was calculated using the formula:tumor volume = (smallest diameter)2 × (largest diameter) × 0.52. The tumor inhibition rate was calculated as follows: tumor inhibition rate (%) = (1 − the tumor weight in treatment group/tumor weight in control group) × 100%. The one-way analysis of variance (ANOVA) was used to determine the statistical significance (*P* < 0.05) between multiple groups. ^∗^*P* < 0.05 for rAd-mIL-28B vs. rAd-EGFP.

## Data Availability

Data are available on reasonable request from the corresponding author.

## Abbreviations

Tregs: Regulatory T cells
GAPDH: Glyceraldehyde-3-phosphate dehydrogenase
TME: Tumor microenvironment
rAd-mIL-28B: Recombinant adenovirus vectors expressing mouse IL-28B
rAd-EGFP: Recombinant adenovirus vectors expressing enhanced green fluorescein protein
CTLA-4: Cytotoxic T lymphocyte antigen 4
TGF-*β*: Transforming growth factor-*β*.
